# Dietary Study of Modern Woodmen of World Sanatorium

**Published:** 1915-10

**Authors:** 


**Dietary Study of Modern Woodmen of World Sanatorium,** Woodmen, Col. C. D. Spivak of Denver in Col. Med. September. The diet list for the week, August 4-10, 1913, showed an average of calories from animal food of 2,667; and from vegetable food 1,425, total 4,092, or about 60% more than is needed by the average healthy person at light labor. The per capita, per diem cost was 52 1/2 cents. (Note: This is about proportionate to the cost of an economic but liberal diet for healthy adults, using about 2500 calories a day. At first thought, it would seem that greater economy would be possible in purchasing on a large scale but the disproportionate amount of animal food, meat, eggs, butter, etc., should be considered).

